# Genome-wide fitness analysis of *Salmonella enterica* reveals *aroA* mutants are attenuated due to iron restriction *in vitro*

**DOI:** 10.1128/mbio.03319-23

**Published:** 2024-09-17

**Authors:** Jessica L. Rooke, Emily C. A. Goodall, Karthik Pullela, Rochelle Da Costa, Nicole Martinelli, Chelsie Smith, Maria Mora, Adam F. Cunningham, Ian R. Henderson

**Affiliations:** 1 Institute for Molecular Bioscience, University of Queensland, Brisbane, Australia; 2 Institute of Immunology and Immunotherapy, University of Birmingham, Birmingham, United Kingdom; Yale School of Medicine, New Haven, Connecticut, USA

**Keywords:** *Salmonella*, TraDIS, *aroA*, iron acquisition, vaccines

## Abstract

*Salmonella enterica* is a globally disseminated pathogen that is the cause of over 100 million infections per year. The resulting diseases are dependent upon host susceptibility and the infecting serovar. As *S. enterica* serovar Typhimurium induces a typhoid-like disease in mice, this model has been used extensively to illuminate various aspects of *Salmonella* infection and host responses. Due to the severity of infection in this model, researchers often use strains of mice resistant to infection or attenuated *Salmonella*. Despite decades of research, many aspects of *Salmonella* infection and fundamental biology remain poorly understood. Here, we use a transposon insertion sequencing technique to interrogate the essential genomes of widely used isogenic wild-type and attenuated *S*. Typhimurium strains. We reveal differential essential pathways between strains *in vitro* and provide a direct link between iron starvation, DNA synthesis, and bacterial membrane integrity.

**IMPORTANCE:**

*Salmonella enterica* is an important clinical pathogen that causes a high number of deaths and is increasingly resistant to antibiotics. Importantly, *S. enterica* is used widely as a model to understand host responses to infection. Understanding how Salmonella survives *in vivo* is important for the design of new vaccines to combat this pathogen. Live attenuated vaccines have been used clinically for decades. A widely used mutation, *aroA*, is thought to attenuate *Salmonella* by restricting the ability of the bacterium to access particular amino acids. Here we show that this mutation limits the ability of *Salmonella* to acquire iron. These observations have implications for the interpretation of many previous studies and for the use of *aroA* in vaccine development.

## INTRODUCTION

*Salmonella enterica* is a globally disseminated organism that can cause infections in both humans and animals. *S. enterica* is a facultative intracellular pathogen spread orofecally and is especially prevalent in areas that have poor sanitation or limited access to clean drinking water (1, 2). *S. enterica* can be broadly split into >2,500 serovars, which differ in genome content and host restriction. There are multiple disease presentations of *S. enterica*, which depend upon the host sensitivity and infecting serovar (3). Some serovars are “generalists” that can infect many hosts (e.g., serovar Typhimurium), while others are specialists that can only infect a specific host, such as serovars Typhi and Paratyphi (4, 5).

*S. enterica* causes disease by actively invading epithelial cells of the intestinal mucosa via the well characterized *Salmonella* Pathogenicity Island 1 (SPI-1) encoded type 3 secretion system (6). Invasive disease occurs when bacteria traverse the epithelial barrier and invade phagocytes at the basal surface of the gut lumen (7, 8). These phagocytes then circulate *Salmonella* to systemic sites through the lymphatic system (7, 9). The dynamics of *Salmonella* infections have largely been elucidated using an Nramp^−/−^ murine model of infection (10). In this model the mice are exquisitely susceptible to intracellular pathogens, with a single bacterium injected intraperitoneally or intravenously inducing death within days. Therefore, to understand adaptive immunity and infection dynamics at later time points, researchers often use attenuated *Salmonella* strains where the infections are less severe and are often cleared by the mice (11, 12).

One popular attenuated strain is termed *S*. Typhimurium strain SL3261, which is an aromatic amino acid auxotroph developed by Hoiseth and Stocker via transposon mutagenesis (13). These researchers used a Tn*10* Tet^R^ transposon to mutate the parent strain *S*. Typhimurium SL1344. The corresponding transposon mutants were screened for tetracycline resistance and aromatic amino acid auxotrophy. The researchers then selected for aromatic amino acid auxotrophy and loss of tetracycline resistance to generate SL3261. The resulting auxotrophy was due to transposon inactivation of the *aroA* gene in the shikimate pathway. Mutants in *aroA* are unable to synthesize chorismate, a molecule that sits at the branch point for many biosynthetic pathways including aromatic amino acid (13) and siderophore biosynthesis (14). SL3261 was shown to be highly attenuated in a susceptible mouse model of infection and provided protection against challenge with the virulent SL1344 strain (13). As a result, several live attenuated *Salmonella* vaccine candidates have been based on the *aroA* mutation. In addition, mutants containing an *aroA* deletion have also been used as a vector to deliver anti-cancer molecules into tumors, and the *aroA* mutation has been used to attenuate a wide variety of other pathogens (15). Despite these strains being used for decades in research, the exact mechanism for attenuation is unclear. Aromatic amino acids are abundant within the host (16), and so it is unlikely that loss of aromatic amino acid biosynthesis is the main driver of attenuation. While *aroA* mutants have increased membrane permeability and susceptibility to membrane acting compounds, including human complement (17), the mechanism for this is also unknown.

Here we sought to understand the molecular mechanism for the attenuation of SL3261. We generated dense transposon-mutant libraries in both *S*. Typhimurium SL3261 and the isogenic parent, *S*. Typhimurium strain SL1344. Analyses of these libraries revealed major differences in genes involved in iron acquisition, DNA synthesis, and cell envelope biogenesis. Importantly, these data indicate that SL3261 is restricted in ferric iron uptake, which is the primary cause of its membrane barrier defects and an attenuated phenotype. In contrast to previous reports, our data reveal that *S*. Typhimurium and *S*. Typhi have a similar preference for ferric iron.

## RESULTS

### Construction of an *S*. Typhimurium strain SL1344 transposon library

**Fig 1:**
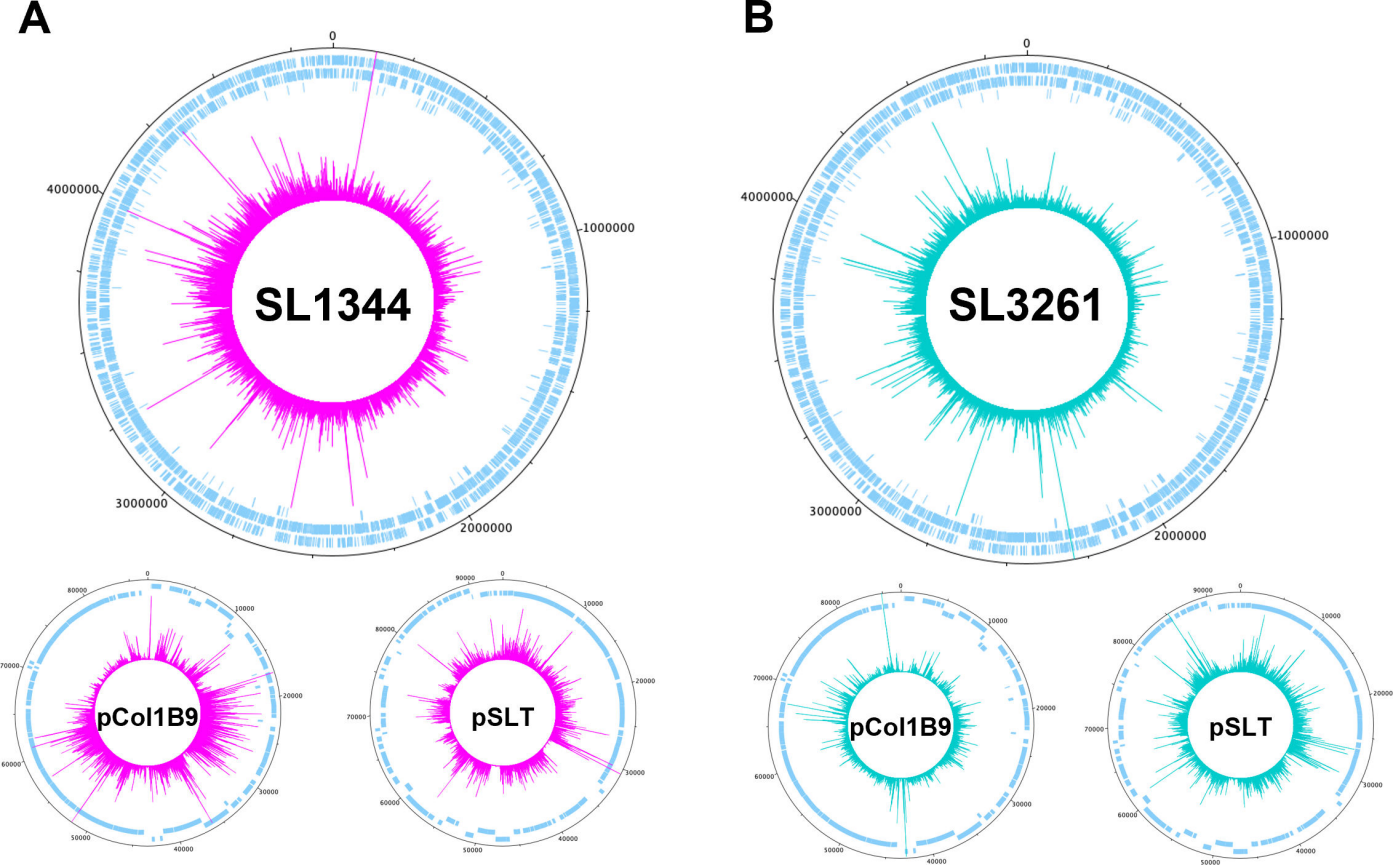
Transposon-mutant libraries in *S*. Typhimurium strains. Chromosome and plasmid maps representing both transposon insertion site location and frequency in *S*. Typhimurium strains (A) SL1344 (pink) and (B) SL3261 (green). Both libraries were constructed via electroporation of an EZ-Tn5 transposon with a kanamycin resistance selection marker (Lucigen) and transformants selected for on Luria-Bertani agar supplemented with 50 µg/mL of kanamycin after 18 h incubation at 37°C.

Transposon-directed insertion-site sequencing (TraDIS) is a transposon insertion sequencing (TIS) method widely used to identify genes required for growth in specific conditions (18–24). To compare the genetic requirement of two *Salmonella* strains with near identical genotypes, we constructed two transposon-mutant libraries. *S*. Typhimurium transposon libraries have been previously constructed in strains SL1344, SL3261, D23580 and 14028s ,(25–29) (Table S1). However, previously published SL1344 libraries were designed for screening in animal models and therefore had a low insertion density; where libraries of sufficient density were described, a catalog of essential genes was not reported. As SL1344 is the parent strain of SL3261, we first constructed a library in *S*. Typhimurium strain SL1344. A library was constructed via transformation with an EZ-Tn*5* transposon and selected on agar plates supplemented with kanamycin (30, 31). Approximately 1.45 million individual colonies were pooled to form the library. To identify the transposon-insertion sites, genomic DNA (gDNA) was extracted from the library pool in duplicate and fragmented. DNA fragments containing the transposon-gDNA junction were amplified and sequenced using an Illumina MiSeq, obtaining >4-M reads per technical replicate. The data were de-multiplexed by barcode and searched for a correct transposon sequence. The barcodes and transposon sequences were trimmed, and the remaining data were mapped to the reference genome (accession NC_016810.1). Altogether, ~10-M reads were successfully mapped for each library using the BioTradis analysis package (Table S2). Some Tn*5* insertion-site preferences have been reported; however, these are considered negligible in the context of an ultra-dense library (30, 32, 33). The transposon insertion sites are distributed around the whole chromosome and plasmids pSLT and pCol1B9 (Fig. 1A). However, we did not identify any transposon insertion events within the pRSF1010 plasmid (Fig. 1A), suggesting this plasmid may not be present in our strain or that it cannot tolerate insertions. The library replicates showed high correlation (Fig. S1) and were pooled for subsequent analysis. Altogether, we identified 625,038 unique insertions in the chromosome and 20,545 and 22,017 unique insertions in the pCol1B9 (NC_017718.1) and pSLT (NC_017720.1) plasmids, respectively. We identified genes required for growth using the BioTraDIS gene_essentiality.R script. Here, we excluded plasmids from our analysis, first, because *S*. Typhimurium SL1344 can be cured of plasmid pSLT (34), suggesting these genes are not required for viability, and second, because of variability in plasmid copy number (between individual cells), this method of analysis can be inaccurate for identifying plasmid genes required for fitness (35). We identified 516 chromosomal genes with significantly fewer transposon insertion events (Table S3). A comparison between *S*. Typhimurium mutant libraries has been previously published (36); therefore, we benchmarked our data against this data set. Of the 516 essential genes in our data set, 495 are protein coding and 363 of these (73.4%) are reportedly required for growth in all other *S*. Typhimurium strains (Fig. S2; Table S3). The proportion of genes unique to our library are consistent with equivalent data sets (34), and variation can be attributed to difference in methodology, conditions, or analysis methods, in addition to strain genotype discussed further below. This served as an important internal control for the validation of our library, and a list of “core” *S*. Typhimurium essential genes are provided in Table S3.

### Construction of an *S*. Typhimurium strain SL3261 transposon library

Following the same protocol outlined above, we constructed a TraDIS library in *S*. Typhimurium strain SL3261. We identified 632,132 unique insertions for the chromosome, 23,431 for pCOL1B9, and 20,194 for pSLT. Like SL1344, we did not obtain any reads mapping to plasmid pRSF1010 (Fig. 1B) (Table S2).

As previously noted, *aroA* encodes the enzyme 3-phosphoshikimate 1-carboxyvinyltransferase that is part of the shikimate pathway. This pathway generates chorismate, which sits as the branch point for many biosynthetic pathways including aromatic amino acids, ubiquinone, and siderophore biosynthesis (Fig. S3A). As expected, given that SL3261 is an *aroA* mutant, no insertions were identified for the *aroA* locus (Fig. S3B). However, we also noticed that there were no insertions at the 5′ end of the downstream *ycaL* gene. We hypothesized that this was possibly due to the way in which the SL3261 strain was constructed (13). Amplification of the *aroA-ycaL* loci (Fig. S3C) and subsequent Sanger sequencing confirmed the deletion of the *aroA* gene and revealed a scar comprising an intact transposase gene that had replaced the 5′ end of *ycaL*, resulting in an in-frame truncation of *ycaL* (Fig. S3D). YcaL is a periplasmic M48 metalloprotease that is involved in outer membrane protein (OMP) biogenesis quality control and interacts with the β-barrel assembly (Bam) complex in *Escherichia coli* (37). M48 metalloproteases are conserved among the tree of life and are important for outer membrane integrity and OMP biogenesis (38). The truncation of *ycaL* in SL3261 resulted in the removal of the lipoprotein signal sequence (Fig. S3E), meaning if the protein was expressed, it would be mis-localized in the cytoplasm and not in the periplasm (Fig. S3F), essentially making SL3261 a double *aroA* and *ycaL* mutant. Therefore, we hypothesized that the loss of YcaL may also contribute to SL3261 attenuation.

### Comparison of differential fitness between *S*. Typhimurium strains

**Fig 2:**
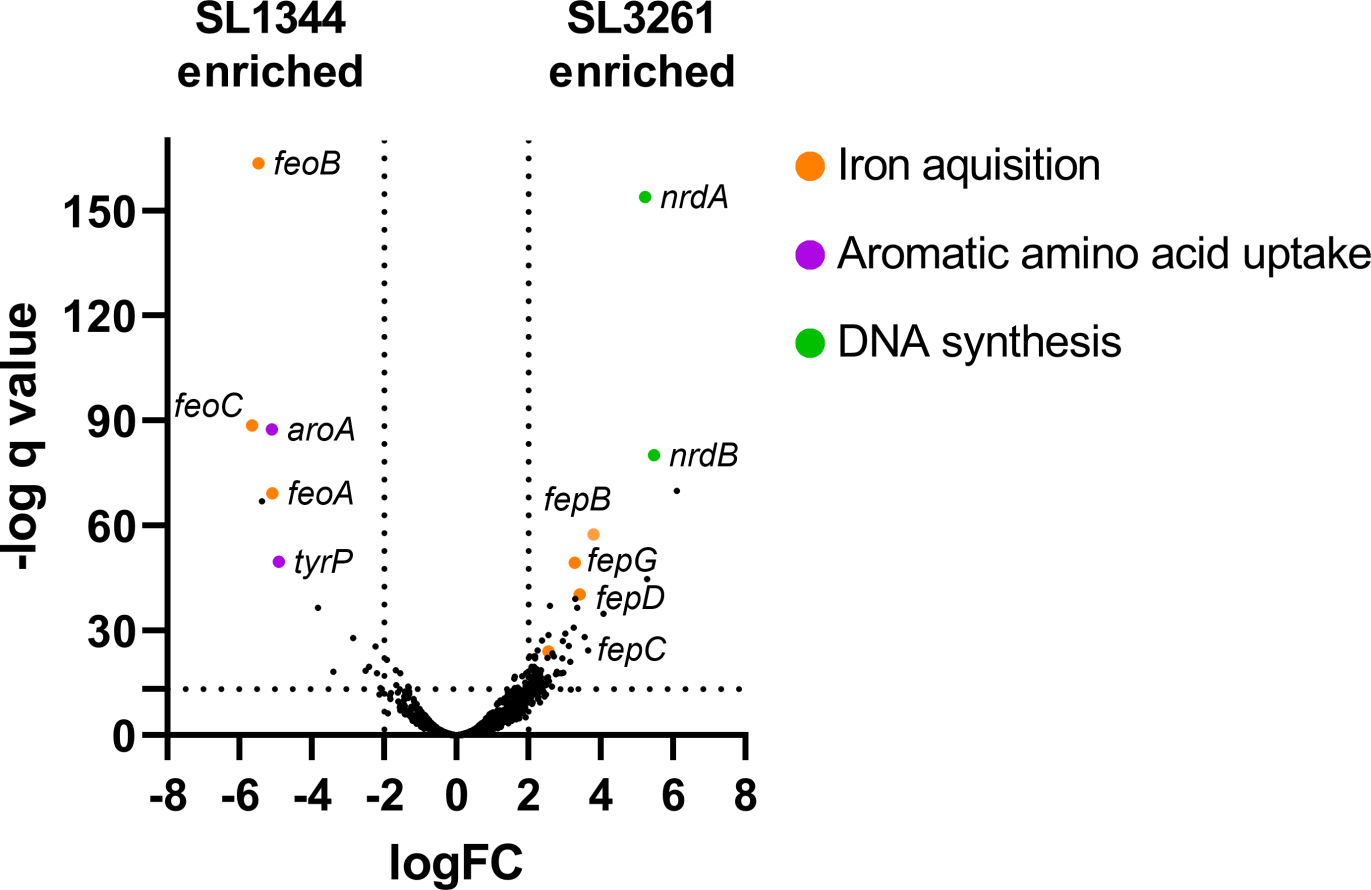
Identification of genes contributing to fitness in each strain. Read counts from each library were compared using edgeR to determine mutant fitness. The data are represented here as a volcano plot where mutants to the right of the *Y* axis are enriched in SL3261, and those to the left are enriched in SL1344.

To identify genes that differentially contribute to fitness in each strain, we used the biotradis tradis_comparison.R script. This method makes use of EdgeR to compare the relative abundance of sequencing read counts per gene for each library as a proxy for mutant abundance (39, 40). Mutants that are “more fit” are expected to be more abundant and therefore have a higher relative read count. Conversely, mutants that are “less fit” will be less abundant and therefore have a lower relative read count. A comparison between the two strains revealed significant differences in genes involved in iron acquisition, DNA biosynthesis, outer membrane biogenesis, and aromatic amino acid import (Fig. 2; Table S4). These data suggest that *S*. Typhimurium strain SL3261 has marked differences in cellular processes in comparison to *S*. Typhimurium strain SL1344, and these are explored further below.

### Aromatic amino acid import

**Fig 3:**
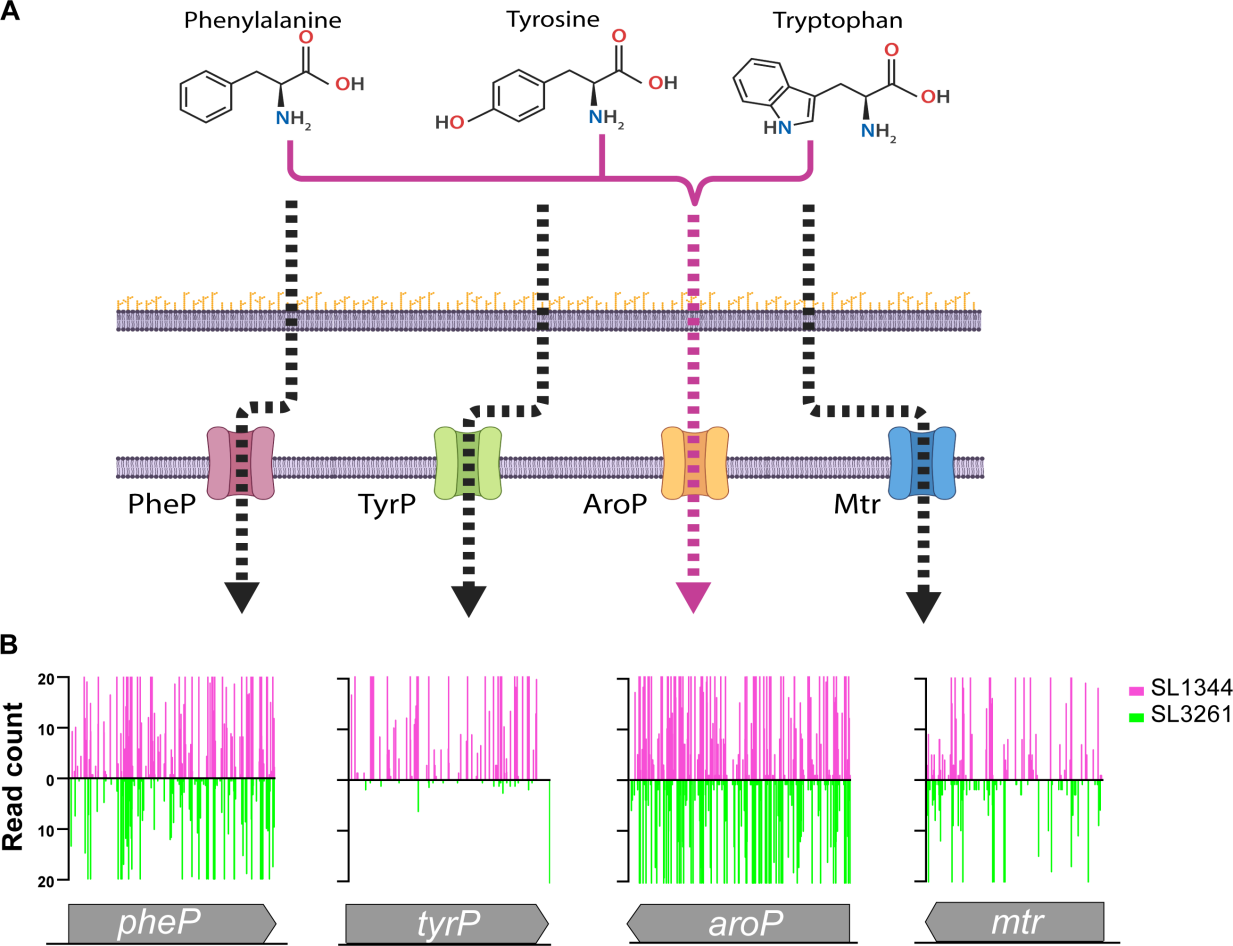
Aromatic amino acid transport mutant fitness. (A) Chemical structure of (left) phenylalanine, (middle) tyrosine, and (right) tryptophan and their importers in the inner membrane. Figure was created with BioRender.com (**B**) Gene insertion profiles of *pheP*, *tyrP*, *aroP*, and *mtr* in SL1344 (pink) and SL3261 (green) TIS libraries. For visualization, reads are capped at 20.

As SL3261 is unable to synthesize chorismate, a precursor to aromatic amino acid biosynthesis, we hypothesized that genes involved in import of aromatic amino acids would be essential. *S. enterica* and *E. coli* have four periplasmic aromatic amino acid permeases that transport aromatic amino acids through the cytoplasmic membrane, specific to each amino acid, using the proton motive force: L-tyrosine is imported by TyrP (41), L-phenylalanine by PheP (41), and L-tryptophan by Mtr (42) and the general aromatic amino acid permease AroP (43) (Fig. 3A). In our TIS data, the permeases for both phenylalanine and tryptophan were non-essential in both SL1344 and SL3261, whereas TyrP, a permease for tyrosine, was essential in SL3261 (Fig. 3B). AroP can transport all three aromatic amino acids (42); thus, our TIS data would suggest one of the following: that under the conditions tested here, AroP cannot or is incredibly inefficient at transport of tyrosine; L-tyrosine is not as abundant as the other aromatic amino acids in Luria-Bertani (LB) agar and thus a specific L-tyrosine transporter is required; or the transporters are differentially regulated under laboratory conditions that result in only TyrP being essential in SL3261.

### Differential fitness of genes involved in iron utilization

Iron is an important metal for life that is involved in various biological processes. Iron exists in two major forms: insoluble ferric (Fe^3+^) and soluble ferrous (Fe^2+^) forms, with ferrous iron considered the more unstable of the two, as it can readily undergo oxidation when exposed to environmental oxygen. The importance of iron import during *Salmonella* infections has been established previously (44–47), and mice that have impaired ability to remove iron from intracellular reservoirs are extremely susceptible to intracellular pathogens (10). The *S. enterica fep* and *feo* operons encode iron acquisition systems for the import of ferric and ferrous iron, respectively (48–50). Ferrous iron can enter the periplasm via outer membrane porins and is transported into the cytoplasm via the FeoAB-YhgG complex (48). In contrast, the import of ferric iron requires secreted siderophores that bind Fe^3+^ directly. The Fe^3+^-siderophore complex is imported through outer membrane porins such as FepA and IroN, a process that requires energy, which is derived from inner membrane complex TonB-ExbB-ExbD (51). Once in the periplasm, the iron-siderophore complex is dissociated and the iron is transported from the periplasm to the cytosol via inner membrane transporter complex FepBCDG. *S. enterica* produces two siderophores: enterobactin and salmochelin.

**Fig 4:**
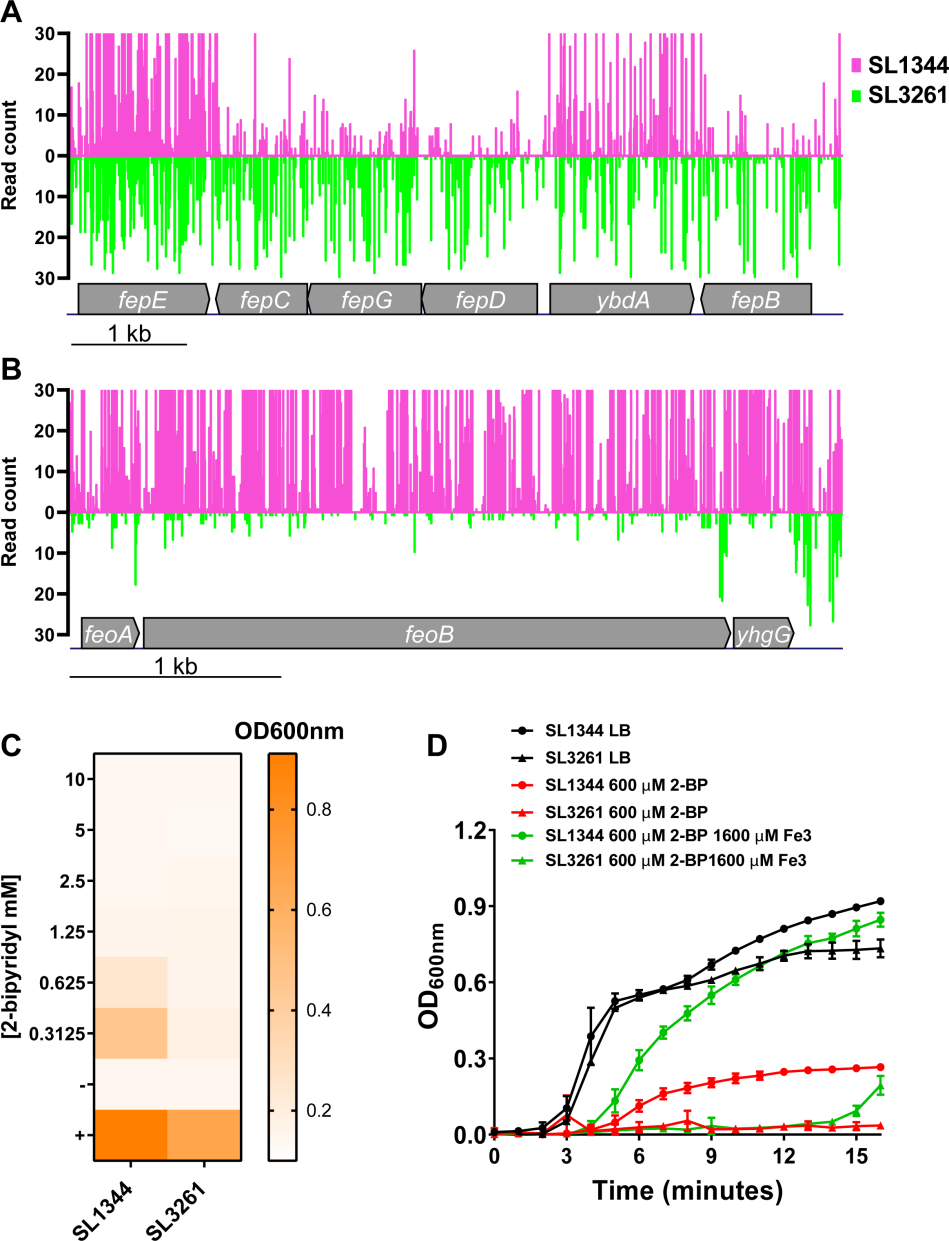
Ferric and ferrous iron importer fitness in SL1344 and SL3261. (**A**) Gene insertion profiles of the *fep* operon and (**B**) *feo* operon. (**C**) End point optical density (OD)_600 nm_ measurements for SL1344 and SL3261 grown in LB medium supplemented with varying concentrations of the iron chelator 2-bypyridyl (2-BP). (**C**) Growth curve of SL1344 and SL3261 in the presence of 2-BP alone or 2-BP supplemented with iron (III).

The fitness of transposon mutants for these iron utilization pathways was different between SL1344 and SL3261: SL1344 preferred import of ferric iron (*fepBCDG*) (Fig. 4A), whereas SL3261 preferred ferrous iron import (*feoAB yhgG*) (Fig. 4B). SL3261 was more sensitive to iron sequestration than SL1344 when we supplemented the LB medium with the iron chelator 2-2-bipyridyl (2-BP) at multiple concentrations (Fig. 4C). The growth of SL3261 in the presence of 2-BP could not be restored by the supplementation of ferric iron into the culture medium, whereas topical addition of ferric iron restored growth of SL1344. These essentiality and growth differences are expected due to Aro mutants being unable to synthesize chorismate, which is a precursor for siderophores that are important for scavenging ferric iron from the environment. When we complemented SL3261 with *aroA*, we restored the ability to withstand 2-BP-mediated ferrous iron sequestration (Fig. S4A through D). However, what was intriguing was the apparent preference observed in iron utilization systems in SL1344. We observed SL1344 *fep* mutants were less fit in our TIS data, and this can be observed in other TIS libraries in *S*. Typhi (26), invasive non-typhoidal *Salmonella* (iNTS) strain D23580 (36), and *E. coli* K-12 (20). These data are intriguing, as presumably, if the systems for siderophore-mediated iron import are absent, strains can utilize the ferrous iron import system instead, and this might not cause a fitness disadvantage. However, our TIS data would suggest that mutants for the *fep* system are less fit *in vitro*, and this is common among many *S. enterica* serovars.

### Ribonucleotide reductase enzyme essentiality

**Fig 5:**
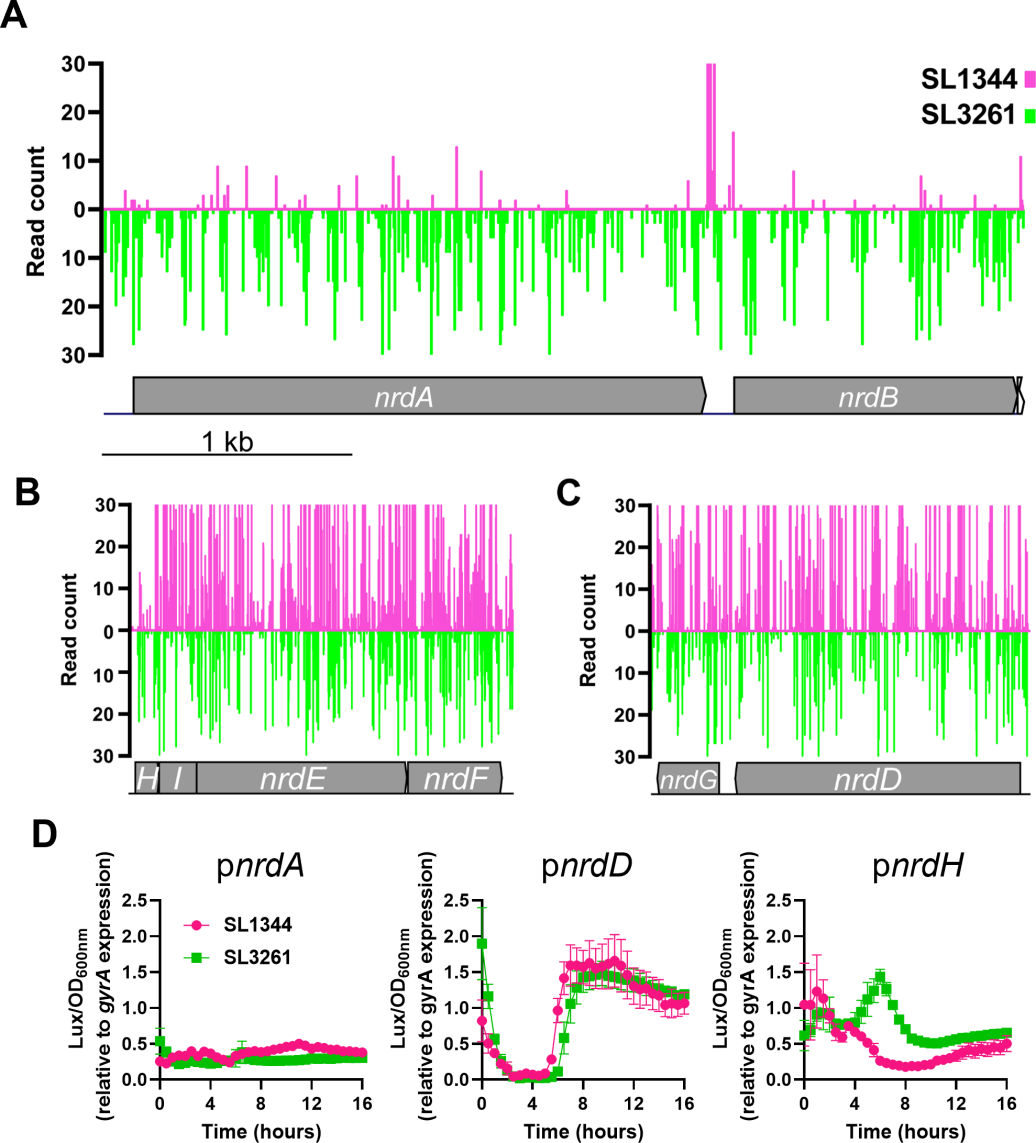
The role of ribonucleotide reductases in the absence of *aroA*. Insertion profiles of (**A**) *nrdAB*, (**B**) *nrdHIEF*, and (**C**) *nrdGD* in the SL1344 (pink) and SL3261 (green) TIS libraries. (**D**) Relative expression of *nrd* genes compared to the *gyrA* promoter over 16 h of growth on LB agar. Data here are from three biological replicates and error bars show the standard error of the mean.

The reduction of nucleotide triphosphates (NTPs) to deoxyribonucleotide triphosphates (dNTPs) is an essential process that occurs in all living organisms, which is achieved by ribonucleotide reductase (RNR) enzymes. There are three classes of RNR enzymes in *S. enterica*, which are differentially regulated (Fig. S5A) (52). The class Ia RNR operon encoded by *nrdAB* is the major RNR class expressed *in vitro* and is essential in *E. coli* and *S. enterica* (20, 26, 36, 53). The class Ib RNR operon is encoded by *nrdHIEF*, and expression is induced by low-iron conditions (54). The class III operon encoded by *nrdDG* is expressed under anaerobic conditions (55). In addition, each RNR enzyme complex requires a metal co-factor, and this is dependent upon the class (56) (Fig. S5A). NrdAB requires Fe^3+^, whereas NrdHIEF require Fe^3+^ or Mn^3+^ and NrdGD require Fe^2+^. Our TIS data revealed that *nrdAB* is not essential is SL3261 (Fig. 5A). Surprisingly, when we interrogated our TIS data for the other RNR operons we observed that none are essential in SL3261 (Fig. 5B and C). A previous TIS study in *S*. Typhimurium suggested the essentiality of *nrdA* was iron dependent as *nrdA* transposon mutants were more fit in the presence of the iron chelator 2-dipyridyl (57, 58). In *E. coli*, *nrdAB* can be deleted provided there is an additional copy of *nrdHIEF* present either on the chromosome or a plasmid (59), suggesting that over expression of one of the other two RNR operons is sufficient to relive *nrdAB* essentiality. Due to the iron dependency of *nrdA* essentiality, differential RNR transcriptional regulation and iron co-factor requirement, we hypothesized that *nrdAB* transposon mutants become more fit in SL3261 due to ferric iron limitation. To overcome the lack of ferric iron and the need for RNR enzyme function, we hypothesized that the expression of either *nrdHIEF* or *nrdGD* would be increased in SL3261 compared to SL1344. To test this hypothesis, we generated luciferase reporter constructs whereby luminescence expression is driven by *nrd* promoters in the vector pLUX as well as a control promoter *gyrA*. We tested the luminescence intensity of each construct in *S*. Typhimurium SL1344 under three conditions: mid-exponential phase (MEP), anaerobic shock and low-iron (Fe) shock (52). We show that the *nrdA* promoter shows similar activity in all conditions tested whereas the *nrdD* promoter was significantly more active under anaerobic conditions and *nrdH* under low iron (Fig. S5B), as expected. These data demonstrate that the reporter constructs respond appropriately in SL1344. To understand whether the *nrd* genes are regulated differently in SL3261, we transformed our reporter plasmids into SL3261 and analyzed the relative luminescence intensity generated by each construct compared to the *gyrA* promoter over 16 h of growth on LB agar, replicating selection conditions of our TIS library construction. We observed that there were no significant differences of either the *nrdA* or *nrdD* promoters between SL1344 and SL3261, but we did observe a different pattern of *nrdH* promoter activity between strains (Fig. 5D). These data suggest that the RNR operons are differentially regulated between strains and that in SL3261, NrdHIEF is expressed at higher levels than in SL1344 at certain points during growth, and this can explain the apparent fitness of NrdAB transposon mutants in this background.

### Fitness of genes involved in outer membrane biogenesis

The Gram-negative bacterial outer membrane is asymmetric and composed of phospholipids at the inner leaflet and lipopolysaccharide (LPS) in the outer leaflet. LPS is an activator of host innate immunity via toll-like receptor 4 signaling (60) and is important for barrier function of the outer membrane (61). In *Salmonella enterica*, LPS is composed of lipid A, inner and outer core oligosaccharides, and O-antigen. In *S. enterica* group B (O4) serotypes such as SL1344 and SL3261, the O-antigen is composed of repeating units of galactose-rhamnose-mannose-abequose sugars (62) (Fig. S6A). The addition of sugar subunits to core oligosaccharide and O-antigen are co-ordinated by multiple enzymes that are arranged in multiple operons (62). Surprisingly, mutations in several genes involved in LPS and O-antigen biosynthesis conferred increased fitness on SL3261 compared to SL1344 (Fig. S6A through D), despite no obvious differences when we compared the LPS profiles of both SL1344 and SL3261 (Fig. S6E). Some of these gene clusters were also identified in a screen for transposon mutants that were differentially fit under iron-restricted conditions (63), suggesting that the differential fitness we observed in our data set could also be attributed to limited iron availability in SL3261.

**Fig 6:**
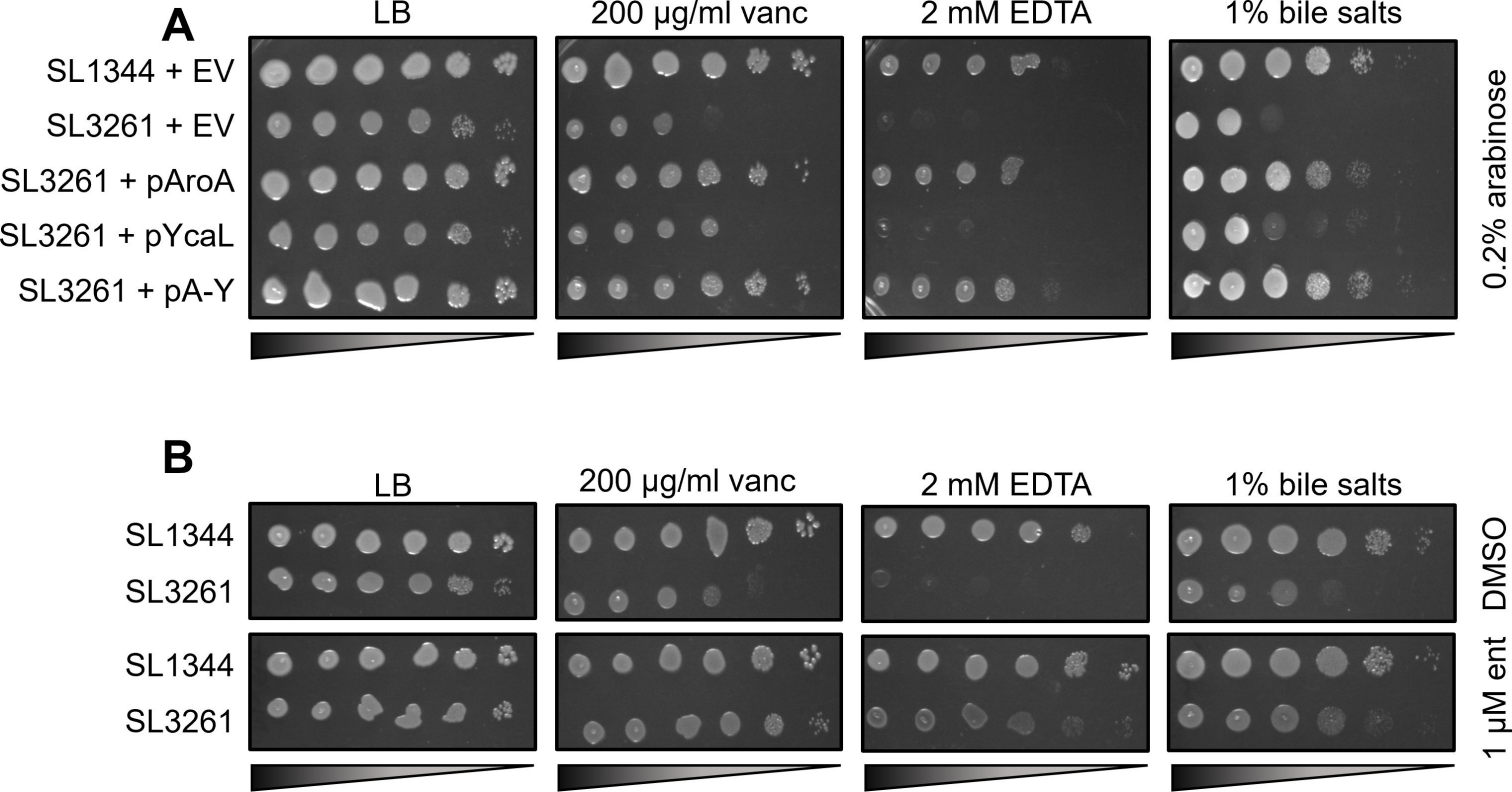
Outer membrane permeability screens for SL1344 and SL3261. (**A**) Overnight cultures of each strain were serially diluted and spot plated onto LB agar supplemented with 0.2% arabinose and either 200-µg/mL vancomycin, 2-mM EDTA, or 1% bile salts. (**B**) Overnight strains serially diluted and plated onto LB agar plates supplemented with the same membrane acting compounds and either Dimethyl Sulfoxide (DMSO) or 1-µM enterobactin. plates were incubated at 37°C for 16–18 h.

Aro mutants are known to have outer membrane permeability defects and to be susceptible to compounds that are membrane acting, such as EDTA, albumin, complement, and bile salts (17). Given that YcaL is purported to interact with the Bam complex during OMP biogenesis, we hypothesized that loss of YcaL in SL3261 may disturb the OM barrier function. To test this hypothesis, we screened our strains and relevant complements on membrane acting compounds: vancomycin, bile salts, and EDTA. We observed that for each compound, complementation of AroA in SL3261 restored membrane barrier permeability, but complementation with YcaL alone did not (Fig. 6A). In conjunction, we generated specific null mutations in both *aroA* and *ycaL* in SL1344 and showed that deletion of *aroA* and not *ycaL* replicated the SL3261 barrier defects, and *aroA* complementation restored barrier function (Fig. S7A). As *aro* mutants are deficient in synthesizing aromatic amino acids and siderophores, we wanted to uncouple the mechanism behind the membrane barrier defect observed. To that end, we repeated our chemical screens with SL1344 and SL3261 and either supplemented the agar with or without 1-µM enterobactin. We observed that enterobactin completely restored barrier function in SL3261 (Fig. 6B) and in SL1344 Δ*aroA* (Fig. S7B), suggesting iron limitation due to loss of AroA causes the barrier defects observed in SL3261.

## DISCUSSION

TIS methodologies have been used to identify essential genomes of many bacterial pathogens (18, 20, 23, 25, 26, 36). Here, we compared the essential gene list of the extensively used laboratory *S*. Typhimurium strain SL1344 with other Typhimurium TIS data sets (36). We revealed a list of 363 Typhimurium genes that are essential in all strains tested. Differences in essential genes can arise due to differences in how libraries were constructed, and how the data were analyzed. For example, our libraries were constructed without salt to increase transformation efficiency, and this might explain some of the differences in our essential gene lists compared to other published data sets.

Here we used TIS to compare transposon-mutant fitness between two model strains of *S*. Typhimurium: SL1344 and SL3261. One of the main findings of this study was that transposon mutants responsible for iron utilization were differentially fit between strains and that iron limitation heavily influenced SL3261 phenotypes. We observed that *S*. Typhimurium SL1344 preferred ferric iron, whereas SL3261 preferred ferrous iron, due to an inability to synthesize siderophores. This apparent preference for ferric iron appears to be conserved among other *Salmonella* serovars, including Typhi. In fact, a previous study concluded that serovars Typhi and Typhimurium prefer utilizing different irons: Typhi with ferric and Typhimurium ferrous, and that this was due to the different niches that these serovars occupy during human infection (26). However, this study did not account for the usage of SL3261 as the Typhimurium strain, and as we have shown here, this strain cannot use ferric iron due to the abolition of the siderophore biosynthetic pathway. Our study and others have now demonstrated that many serovars of *Salmonella* prefer ferric iron and this is not limited to Typhi, suggesting that iron utilization does not differ between serovars or infectious niches.

SL3261 is highly attenuated in mice, and *aroA* mutants have been identified as attenuating in multiple serovars of *Salmonella* and other bacterial pathogens (64–67). These data suggested that synthesizing aromatic amino acids was important *in vivo* and that perhaps these amino acids were in short supply in the host. However, recent data suggests that aromatic amino acids are not restricted inside the host and that mutants in genes that are dedicated to making aromatic amino acids are not attenuated (25). Given that *aroA* mutants are unable to synthesize chorismate, which sits at the branchpoint for at least three different biosynthetic pathways, these data suggest that the cause of attenuation in *aro* mutants was likely due to functions other than aromatic amino acid auxotrophy. In addition, *aroA* was determined to be important for *S*. Typhimurium colonization on alfalfa sprout roots, and this attenuation could not be rescued by topical addition of neither tryptophan nor phenylalanine (68). This study also demonstrated that topical addition of ferrous iron could restore root colonization, and it was concluded that the lack of siderophore production and not the lack of aromatic amino acids was important for *S*. Typhimurium colonization of alfalfa roots (68).

Chorismate is a precursor for both ubiquinone, an electron carrier, and para-amino benzoic acid, a precursor to folate. A study by Felgner and colleagues demonstrated that loss of *aroA* increased membrane permeability, including sensitivity to EDTA (69). This study also determined that ubiquinone mutants did not phenocopy *aroA* mutants in immunogenicity during murine infections nor in *in vitro tests*, suggesting that even though chorismate is a precursor to ubiquinone, lack of ubiquinone alone could not explain an *aroA* mutant phenotype (69).

Genes involved in ferric iron import are known to be important for infections (46), and the *fep* operon is upregulated inside macrophages and the cytosol of host cells (45, 52). There is also evidence that *nrdHIEF* is upregulated in host cells, suggesting iron limitation, and that during infections, the major RNR enzymes used to synthesize DNA are not NrdAB and therefore are different from standard laboratory conditions (45, 52, 70). We also know that susceptible mouse strains lacking a functional copy of the protein Nramp1 are incredibly susceptible to intracellular infections, including *Salmonella* (10). *Nramp1* encodes a divalent cation transporter that can remove iron from host cell vacuoles and stimulate lipochalin-2 expression, a host iron chelator, thereby reducing intracellular iron concentrations (71, 72). In the absence of Nramp1, murine macrophages are unable to limit intracellular iron as efficiently, and therefore, these macrophages are more susceptible to infection. Mouse strains considered resistant to *S. enterica* infections have a functional copy of Nramp1, and therefore, the reason for the resistance to *Salmonella* infections is because these mice can efficiently restrict intracellular iron concentrations.

Gene mutations that cause membrane barrier defects can cause *S*. Typhimurium to become susceptible to membrane targeting compounds such as cationic anti-microbial peptides and detergents, such as bile salts, resulting in attenuation during infection (73). Aro mutants are known to have membrane barrier defects, and here we show that barrier function can be restored in SL3261 by complementation of AroA on a plasmid and supplementation of enterobactin. As enterobactin supplementation completely restored barrier function in SL3261, we stipulate that in addition to iron limitation, SL3261 would experience severe membrane defects *in vivo*. This additional attenuating phenotype would contribute to the severe phenotype observed during SL3261 infections.

During this study, we identified that SL3261 is essentially a double mutant of *ycaL* and *aroA*. YcaL is a metalloprotease that interacts with the Bam complex to facilitate OMP folding (37). Here, we could not identify any mutant fitness defects that could be obviously, or experimentally, attributed to the loss of YcaL. High-throughput screens have also failed to find an obvious phenotype for YcaL in *E. coli* (74). However, roles for homologs have been identified, and a role for YcaL may not be evident under the lab conditions tested in our study.

Overall, our data reveal the power of high-resolution TIS screens for understanding the contribution of genes to the overall fitness of a strain. We have revealed for the first time a direct link between iron availability and OM stability, potentially paving the way for future research into novel complementary therapeutic interventions to combat the rise of anti-microbial-resistant infections.

## MATERIALS AND METHODS

### Media and growth conditions

Strains and plasmids used in this study are listed in Table S5. All strains were grown in LB broth at 37°C with shaking unless stated otherwise. LB agar was made using 1.5% agar in LB. M9 minimal media was prepared using 5× M9 salts (Sigma Aldrich) and supplemented with 200-mM MgSO_4_, 1-mM CaCl2, 30-µg/mL L-histidine, and 0.4% (wt/vol) glucose. Growth curves were completed in 96-well plates, and optical density (OD)_600 nm_ was measured using a Clariostar plate reader.

### Molecular biology

Primers used in this study are listed in Table S6. pQE60*NdeI* vectors (75) were generated using Gibson assembly (76). Briefly, the pQE60*NdeI* backbone was amplified by PCR using primer pair pQE60_ndeI_GA_F and pQE60_ndeI_GA_R. The aroA-his and aroA-ycaL-his inserts were also generated by PCR, with the primers including 20- to 30-bp homology with the target vector. Insert and vector PCR fragments were incubated with Gibson Assembly mastermix (NEB) for 1 h at 50°C. The assembled vectors were then transformed into NEB ultra-competent *E. coli* DH5α cells using heat shock. Inserts were subcloned into pBAD18 using the *EcoRI* or *SalI* and *HindIII* restrictions sites. For cloning into pLUX (77) , promoter regions from target genes were cloned into the *XhoI* and *BamHI* restrictions sites. All transformants were confirmed by PCR and Sanger sequencing.

Null deletion strains were generated using the Datsenko and Wanner approach (78). Briefly, linear fragments containing the *aph* gene flanked by regions of homology to the SL1344 chromosome were transformed into cells expressing λ-red recombinase. Resulting mutants were screened on agar plates containing kanamycin and confirmed by PCR. The *aph* gene was removed by the addition of the plasmid pCP20 encoding the FLP recombinase. Null deletions were confirmed by plating on agar plates and by PCR.

### Protein sample preparation, SDS-PAGE, and Western blotting

Approximately 10^9^ colony-forming units (CFU) from overnight cultures were centrifuged and resuspended in 2× Leammli sample buffer (Sigma). The samples were boiled and loaded onto 4%–12% NuPage gels. After proteins were separated by SDS-PAGE electrophoresis, proteins were transferred to nitrocellulose membranes using a TurboBlot transfer system and were blocked in 5% skim milk. Membranes were probed with either anti-RNAp antibodies (1:2,000) or anti-his antibodies (1:5,000). Secondary goat anti-mouse IgG IRDye 680RD antibodies were used to detect protein bands, and the blots were imaged using an Odyssey CLx machine.

### TraDIS library construction

Both the SL1344 and SL3261 transposon libraries were constructed using the following method. Electrocompetent cells were generated after growth at 37°C in 2xTY broth (12-g tryptone, 6-g yeast extract, and 3.4-mM CaCL2 in 1 L) without salt until an OD_600_ of 0.6. Cells were pelleted at 5,000× *g* and washed in ice-cold sterile distilled water. The final cell pellet was resuspended in iced-cold 10% glycerol (vol/vol). Aliquots of cells were mixed with EZ-Tn5 <KAN-2> transposome (Lucigen) and incubated on ice for 30 min. Cells were pulsed at 2,500 V, and pre-warmed Super Optimal broth with Catabolite repression (SOC) medium was immediately added to the sample for recovery. Cells were incubated at 37°C for 2 h and transposon mutants were selected on LB agar plates supplemented with 50-µg/mL kanamycin. Plates were incubated at 37°C for 18 h. Colonies were scraped from each plate and pooled to form the library, stored in a single falcon with 30% LB-glycerol.

### Transposon junction sequencing

Genomic DNA was extracted from each library using a RTP Stratec DNA extraction kit following the kit protocol. DNA was fragmented using a Covaris bioruptor to achieve a fragment size of ~200 bp. The fragments containing the transposon junctions were amplified by PCR and prepared for sequencing using the NEB Ultra I kit (New England Biolabs) following the manufacturer’s instructions. The final sample was quantified by qPCR using a Kapa Library Quantification kit for Illumina platforms. Samples were sequenced using an Illumina MiSeq, obtaining ~5 million reads per sample. Data are deposited to the European Nucleotide Archive (accession number PRJEB65934).

### Sequencing data analysis

The data were processed first to remove any barcode using the fastx barcode splitter and trimmer tools from the Fastx toolkit. Sequence data with a correct barcode and transposon sequence, following trimming, were processed using Bio-Tradis and subsequently mapped to the SL1344 reference genome (accession numbers NC_016810.1, NC_017718.1, NC_017719.1, and NC_017720.1). Comparative analyses between libraries were also done using the Bio-TraDIS tradis_comparisons.R script, using a minimum read count of 100 for comparison and 5% trim applied to both the 5′ and 3′ end of each gene. Data were inspected in the Artemis genome browser (79). The data can also be visualized in our online browser, available at https://tradis-vault.qfab.org/apollo/jbrowse.

### Growth in iron-limiting conditions

LB medium was depleted of iron using the iron chelator 2-2bipyridyl. A concentration gradient was generated in a 96-well plate, and bacteria were added to a starting OD_600 nm_ of approximately 0.02. Growth (OD_600 nm_) was measured every 30 min for 16 h in a polarstar plate reader. For iron supplementation growth, iron was chelated using 600 µm of 2-2 bipyridyl and Fe^3+^ supplementation at 1.6 mM.

### Screening for outer membrane defects

Overnight cultures were diluted to approximately 10^9^ CFU/mL and serially diluted prior to spotting onto LB agar plates supplemented with either DMSO, 1 µM enterobactin, 200 µg/mL vancomycin, 2 mM EDTA or 1% bile salts (w/v). Plates were incubated overnight at 37°C for 16–18 h.

### Reporter screening

Testing promoter-reporters were completed under MEP, low iron, and anaerobic conditions as previously described (80). Bacteria were added to black-walled 96-well plates, and luminescence was measured in a Tecan plate reader and normalized to OD_600 nm_. For reporter activity on agar, 100 µL of LB agar was added to black-walled 96-well plates, and 10^6^ CFU was inoculated into each well. OD_600 nm_ and luminescence were measured in a Clariostar plate reader.

### LPS preparations

Approximately 10^9^ CFU of overnight cultures was pelleted and resuspended in Laemmli buffer. Cells were boiled prior to the addition of proteinase K and incubated for 1 h at 60°C. Proteinase K was heat inactivated prior to analysis by SDS-PAGE and silver staining.

## AFTER EPUB

*[This article was published on 17 September 2024 with errors in the title. The title was corrected in the current version, posted on 19 September 2024.]*

## SUPPLEMENTAL MATERIAL

Supplemental figures mbio.03319-23-s0001.docx Fig. S1 to S7.

Table S1 mbio.03319-23-s0002.docx Transposon libraries constructed in *S*. Typhimurium strains

Table S2 mbio.03319-23-s0003.docx Metrics for the TIS libraries generated in this study.

Table S3 mbio.03319-23-s0004.xlsx *S*. Typhimurium essential genes comparisons.

Table S4 mbio.03319-23-s0005.xlsx Mutant fitness comparisons.

Table S5 mbio.03319-23-s0006.docx Strains and plasmids used in this study.

Table S6 mbio.03319-23-s0007.docx Primers used in this study.

ASM does not own the copyrights to Supplemental Material that may be linked to, or accessed through, an article. The authors have granted ASM a non-exclusive, world-wide license to publish the Supplemental Material files. Please contact the corresponding author directly for reuse.
