# Supplementary material for: Genome-wide fitness analysis of Salmonella enterica reveals aroA mutants are attenuated due to iron restriction in vitro
Source: mBio. 2024 Sep 17;15(10):e03319-23. doi: 10.1128/mbio.03319-23 (PMC11481492; doi:10.1128/mbio.03319-23)
Supplement: Supplemental figures — Fig. S1 to S7. [file mbio.03319-23-s0001.docx]

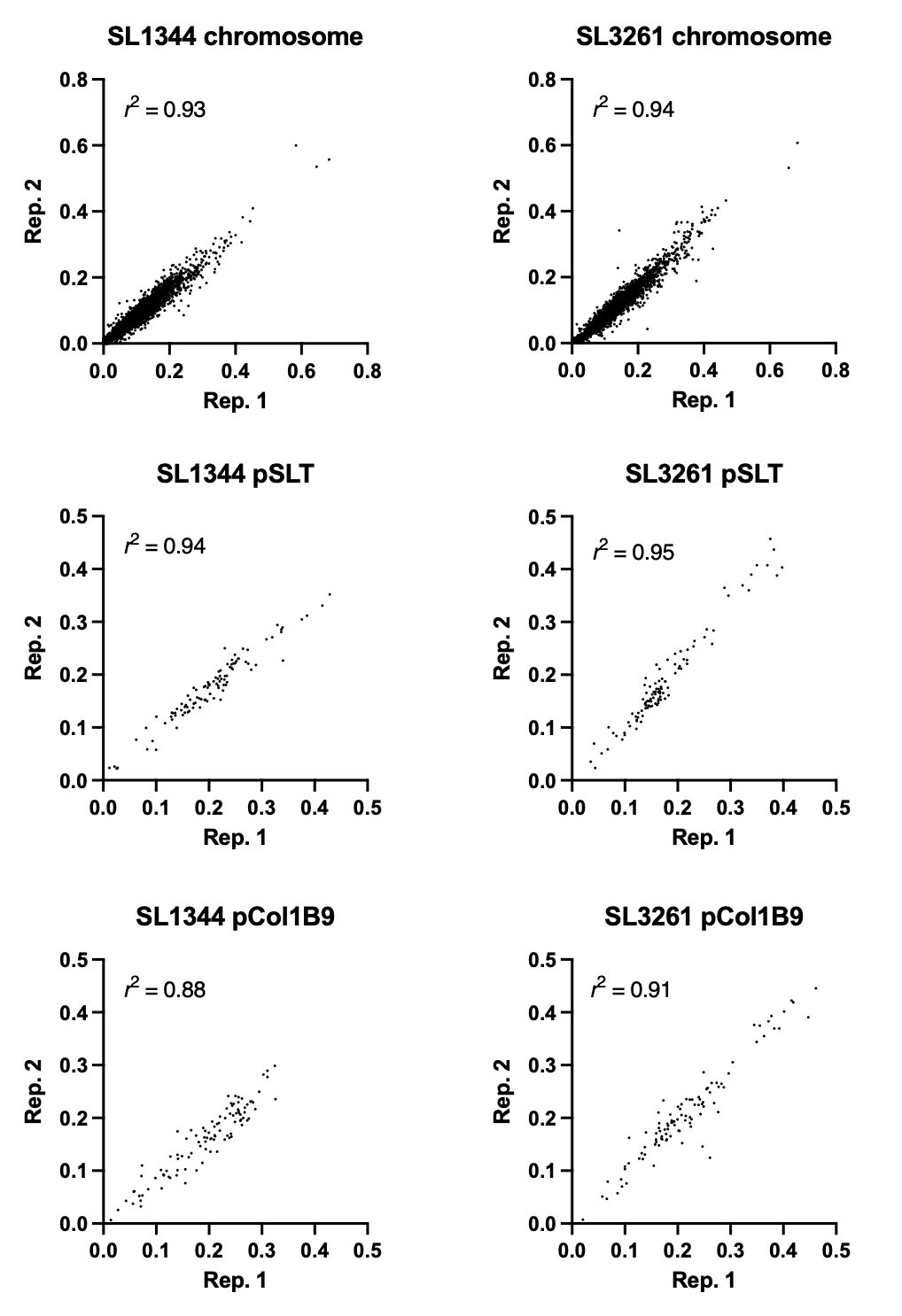
**Supplementary Figures**

**Figure. S1 Comparison of technical replicates.** Scatter graphs showing the insertion index scores of each technical replicate for SL1344 (left) and SL3261 (right) chromosome (top), pSLT (middle) and pCOL1B9 (bottom) plasmids.


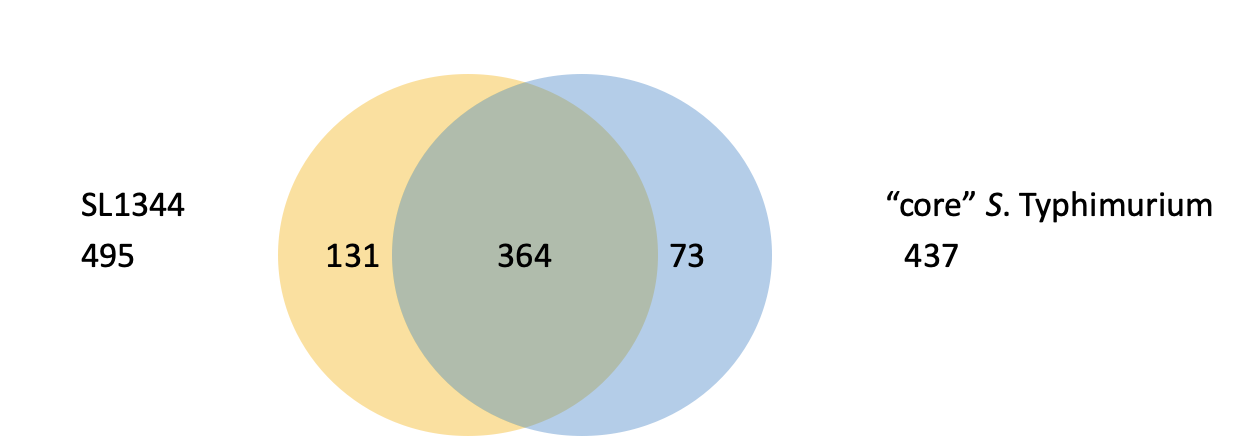


**Figure S2. S. Typhimurium essential gene comparisons.** Venn diagram over essential SL1344 genes identified in this study compared to a compendium of S. Typhimurium essential genes collated from Canals *et al*., 2019.

**
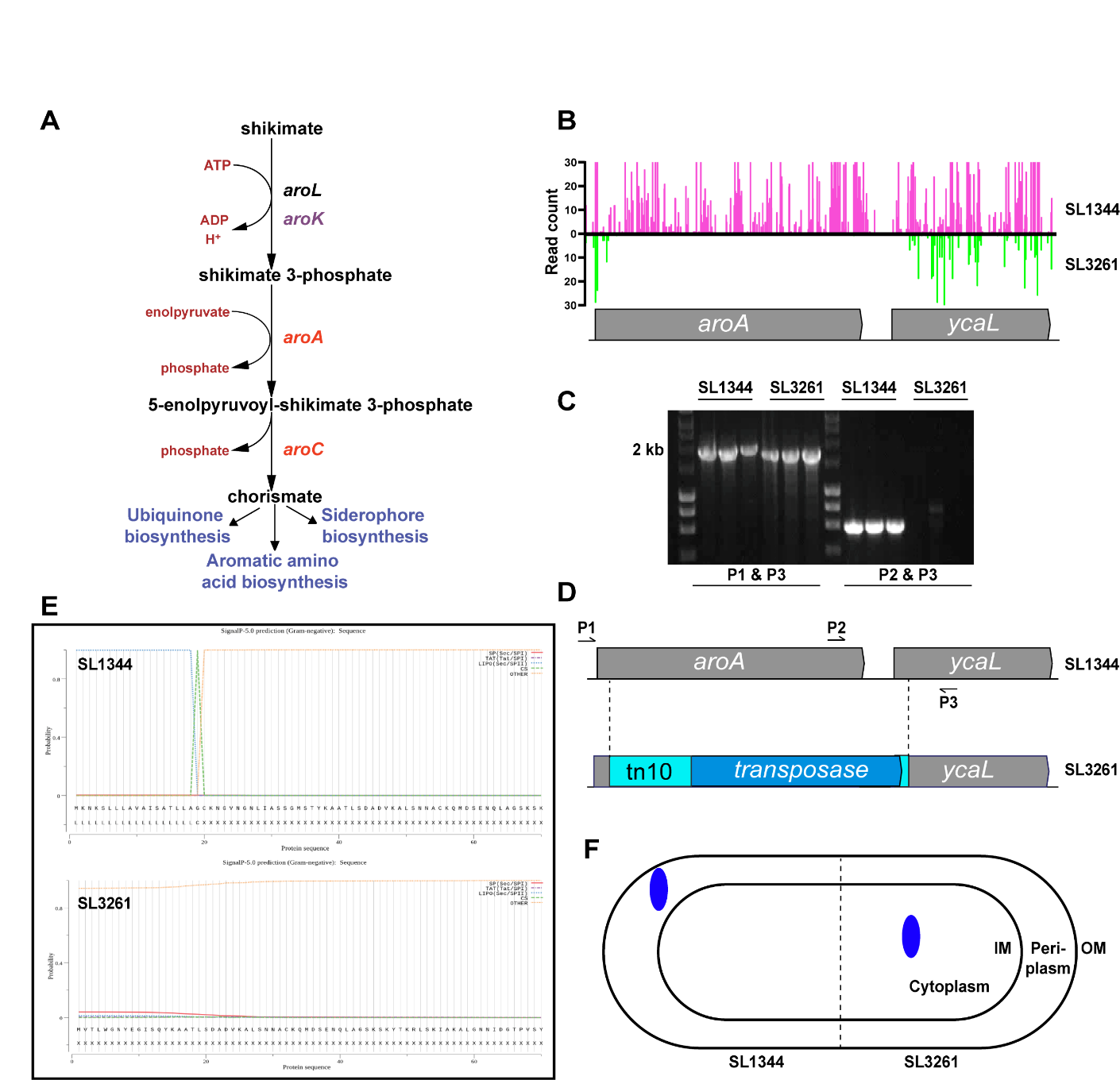
**

**Figure S3. AroA-YcaL loci in *S*. Typhimurium.** (A) Shikimate pathway in *S*. Typhimurium. (B) Insertion plot files for the *aroA-ycaL* loci in S. Typhimurium SL1344 (pink) and SL3261 (green) TIS libraries. (C) PCR amplification of the *aroA-ycaL* loci in SL1344 and SL3261. (D) Schematic of the genomic scar left by the tn10 transposon in SL3261. (E) SignalP plots for the YcaL amino acid sequence in SL1344 (top) and SL3261 (bottom). (F) Schematic of predicted YcaL protein localisation in both SL1344 and SL3261.

**
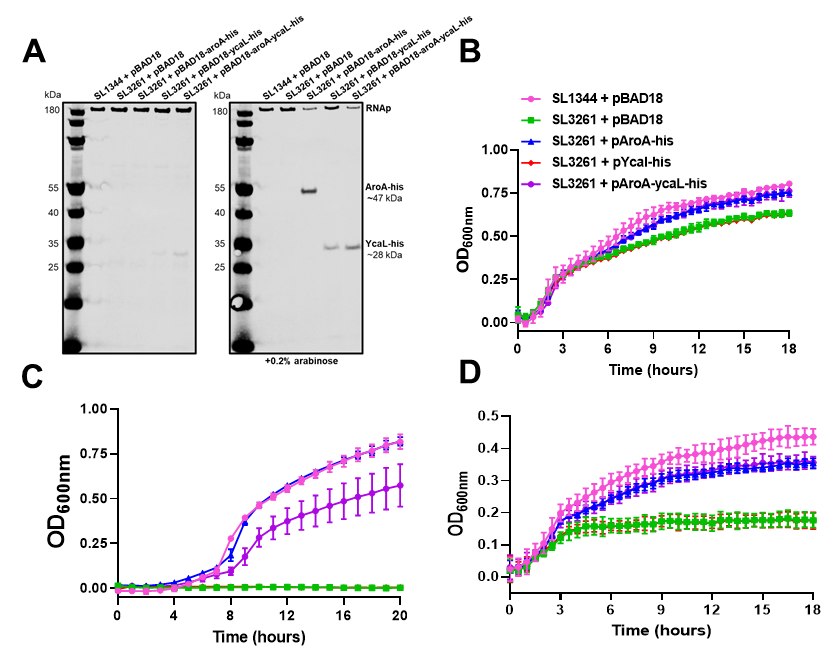
**

**Figure S4. Complementation plasmids in SL3261.** (A) Western immunoblot using anti-poly Histidine tag and anti- RNA polymerase antibodies. Whole cell protein samples were separated on 6-12 NuPAGE gels prior to Western blotting. Images were detected using fluorescent secondary antibodies. Growth of strains in (B) LB medium, (C) M9 minimal medium supplemented with 0.4% glucose and (D) LB medium with 600 µM 2-2 bipyridyl.

**
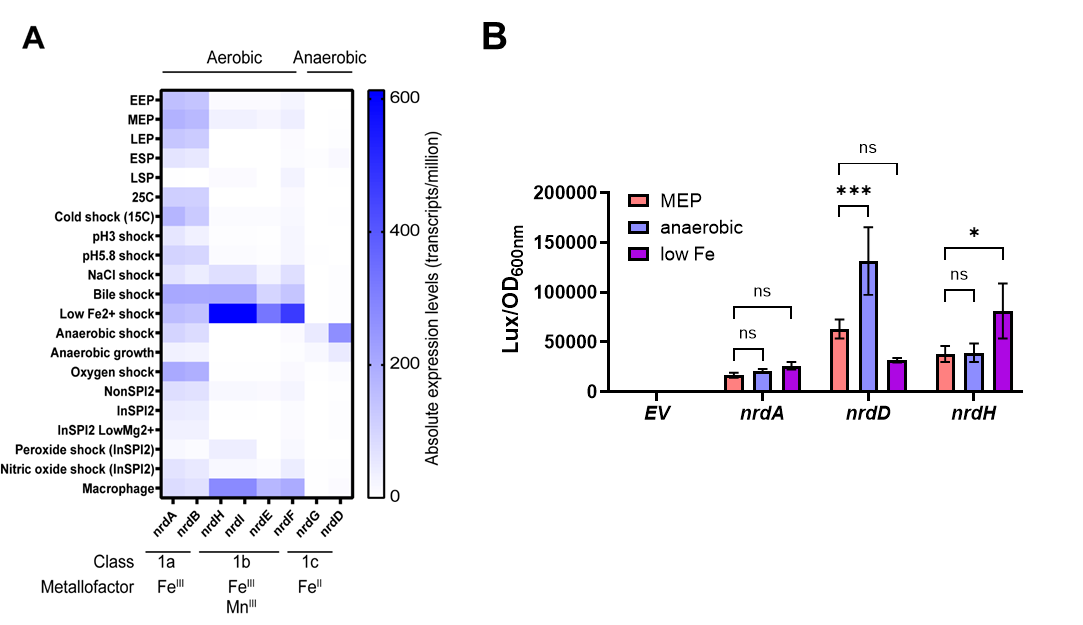
**

**Figure S5. Differential regulation of S. Typhimurium RNR genes.** (A) Absolute expression levels of NrdAB, NrdHIEF and NrdGD under multiple conditions determined by RNA-seq (49). (B) Nrd- luminescence reporter construct activity in mid exponential phase (MEP), anaerobic and low iron conditions.


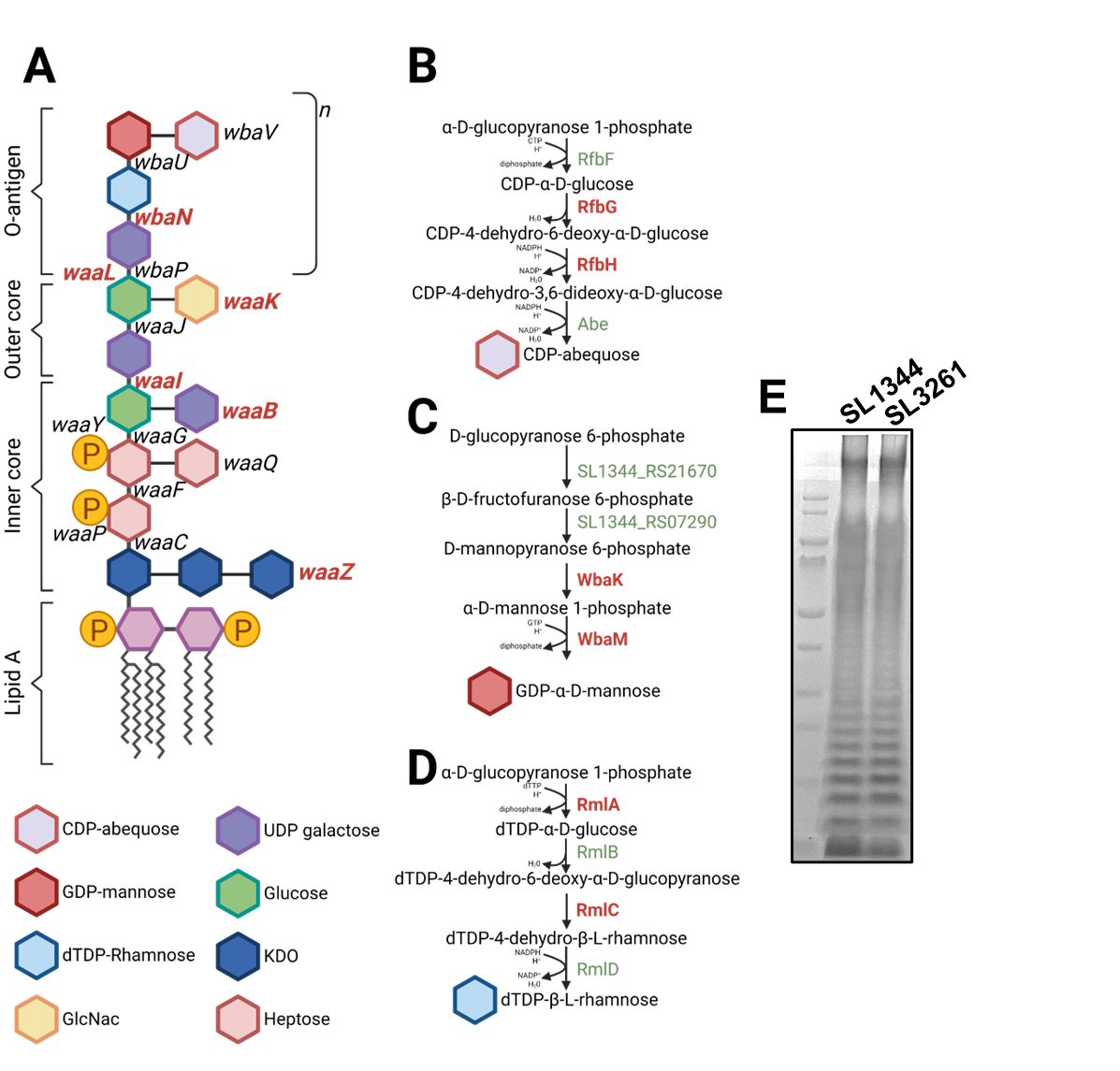


**Figure S6. Mutant fitness of genes involved in LPS biosynthesis.** Enzymes highlighted in red were identified in this study as having transposon mutants that were more fit in SL3261. (A) LPS structure and enzymes involved in the addition of each sugar onto the LPS structure. Sugar-nucleotide biosynthetic pathways for o-antigen sugar units (B) CDP-abequose, (C) GDP-α-mannose and (D) dTDP-β-rhamnose. (E) LPS profiles of SL1344 and SL3261.


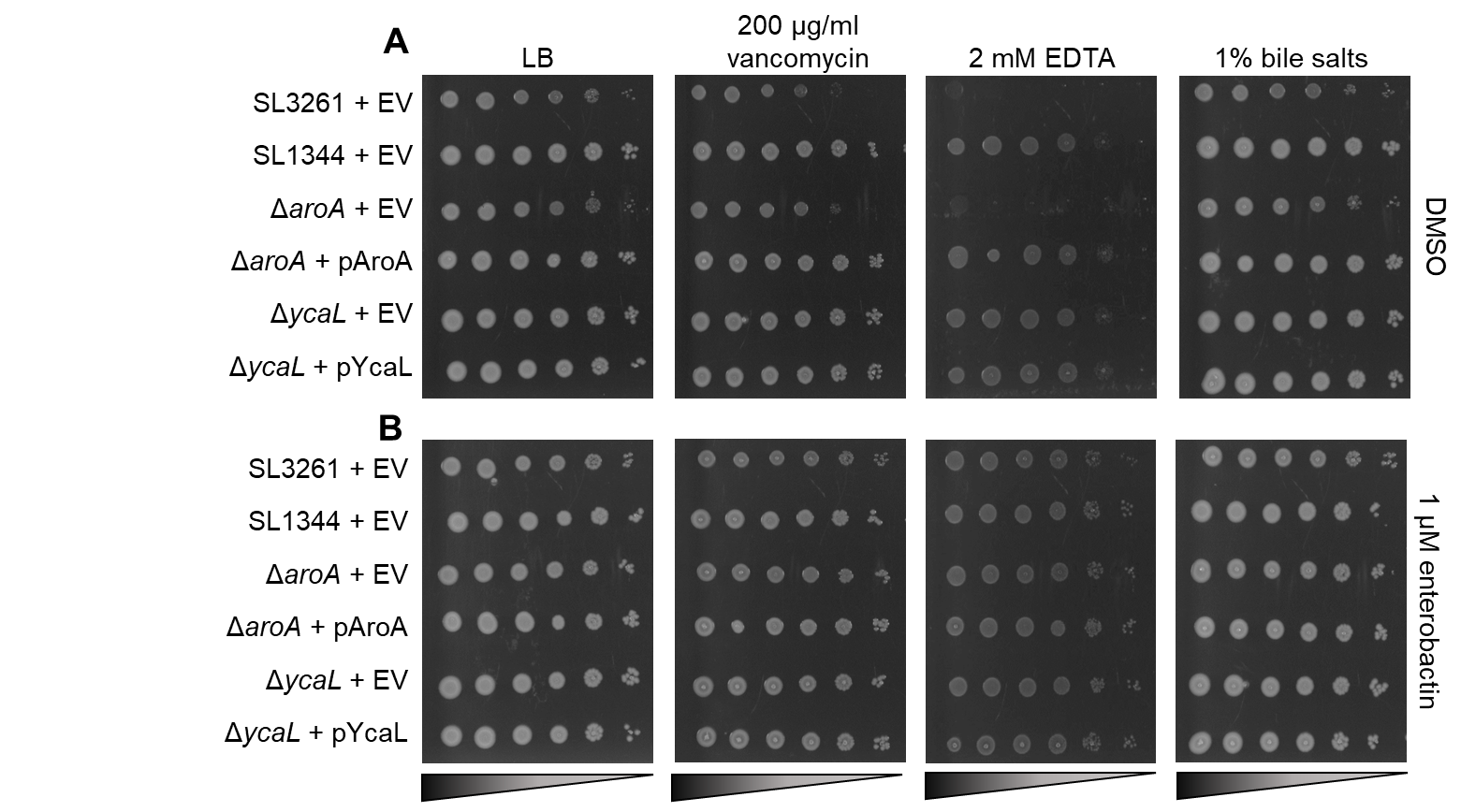


**Figure S7. Outer membrane permeability of *S.* Typhimurium strains. (A)** Overnight cultures of each strain were serially diluted 1:10 and spotted onto agar plates supplemented with 50 µg/ml carbenicillin, 0.05% arabinose, DMSO and the addition of either 200 µg/ml vancomycin, 2 mM EDTA or 1% (w/v) bile salts. (B) Same as A but with the addition of 1 µM enterobactin.
