# Supplementary material for: Genome-wide fitness analysis of Salmonella enterica reveals aroA mutants are attenuated due to iron restriction in vitro
Source: mBio. 2024 Sep 17;15(10):e03319-23. doi: 10.1128/mbio.03319-23 (PMC11481492; doi:10.1128/mbio.03319-23)
Supplement: Table S1 — Transposon libraries constructed in S. Typhimurium strains [file mbio.03319-23-s0002.docx]

**Table S1.** Transposon libraries constructed in *S.* Typhimurium strains**.**

| **Strain** | **Transposon** | **Year** | **No. of mutants**^a^ | **No. UIP^b^** | **Insertion density^c^** | **Reference** |
| --- | --- | --- | --- | --- | --- | --- |
| SL1344 | Tn5/Mu | 2009 | ~10,000 | Not stated | N/A | (1) |
| SL1344 | Tn5 | 2016 | 4,381 | Not stated | N/A | (2) |
| SL1344 | Mu | 2016 | 4,975 | Not stated | N/A | (2) |
| SL3261 | Tn5 | 2013 | ~930,000 | 549,086 | 9 bp | (3) |
| D23580 | Tn5 | 2019 | Not stated | 797,000 | 6 bp | (4) |
| 14028s | Tn5 | 2017 | 325,000 | Not stated | N/A | (5) |

^a^ The number of colonies used to construct the library.

^b^ The number of unique insertion points (UIP) for the library if reported.

^c^ Insertion density is the genome size divided by the number of insertions or mutants reported for that genome to give an approximation of 1 insertion every X bp as a measure of overall library density.

**References**

1. Chaudhuri RR, Peters SE, Pleasance SJ, Northen H, Willers C, Paterson GK, Cone DB, Allen AG, Owen PJ, Shalom G, Stekel DJ, Charles IG, Maskell DJ. 2009. Comprehensive identification of *Salmonella enterica* serovar typhimurium genes required for infection of BALB/c mice. PLoS Pathog 5:e1000529.

2. Grant AJ, Oshota O, Chaudhuri RR, Mayho M, Peters SE, Clare S, Maskell DJ, Mastroeni P. 2016. Genes Required for the Fitness of *Salmonella enterica* Serovar Typhimurium during Infection of Immunodeficient gp91-/- phox Mice. Infect Immun 84:989-997.

3. Barquist L, Langridge GC, Turner DJ, Phan MD, Turner AK, Bateman A, Parkhill J, Wain J, Gardner PP. 2013. A comparison of dense transposon insertion libraries in the *Salmonella* serovars Typhi and Typhimurium. Nucleic Acids Res 41:4549-64.

4. Canals R, Chaudhuri RR, Steiner RE, Owen SV, Quinones-Olvera N, Gordon MA, Baym M, Ibba M, Hinton JCD. 2019. The fitness landscape of the African *Salmonella* Typhimurium ST313 strain D23580 reveals unique properties of the pBT1 plasmid. PLoS Pathog 15:e1007948.

5. Karash S, Liyanage R, Qassab A, Lay JO, Jr., Kwon YM. 2017. A Comprehensive Assessment of the Genetic Determinants in Salmonella Typhimurium for Resistance to Hydrogen Peroxide Using Proteogenomics. Sci Rep 7:17073.
