# Supplementary material for: Genome-wide fitness analysis of Salmonella enterica reveals aroA mutants are attenuated due to iron restriction in vitro
Source: mBio. 2024 Sep 17;15(10):e03319-23. doi: 10.1128/mbio.03319-23 (PMC11481492; doi:10.1128/mbio.03319-23)
Supplement: Table S2 — Metrics for the TIS libraries generated in this study. [file mbio.03319-23-s0003.docx]

**Table S2.** Metrics for the TIS libraries generated in this study**.**

| **Sample** | **Replicate 1** | **Replicate 2** | **Combined** | **UIPs** |
| --- | --- | --- | --- | --- |
| SL1344 chromosome | 506,930 | 431,294 | 625,038 | 7.8 bp |
| SL1344 pCol1B9 | 16,882 | 13,834 | 20,545 | 4.2 bp |
| SL1344 pSLT | 18,120 | 15,723 | 20,017 | 4.7 bp |
| SL3261 chromosome | 534,096 | 440,335 | 632,132 | 7.7 bp |
| SL3261 pCol1B9 | 18,250 | 17,772 | 23,431 | 3.7 bp |
| SL3261 pSLT | 15,496 | 16,371 | 20,194 | 4.6 bp |
