# Supplementary material for: Genome-wide fitness analysis of Salmonella enterica reveals aroA mutants are attenuated due to iron restriction in vitro
Source: mBio. 2024 Sep 17;15(10):e03319-23. doi: 10.1128/mbio.03319-23 (PMC11481492; doi:10.1128/mbio.03319-23)
Supplement: Table S5 — Strains and plasmids used in this study. [file mbio.03319-23-s0006.docx]

**Table S5.** Strains and Plasmids used in this study

| **Strains** | **Description** | **Reference** |
| --- | --- | --- |
| *S.* Typhimurium SL1344 | Histidine auxotrophic variant *S*. Typhimurium | (1) |
| *S.* Typhimurium SL3261 | *aroA* deficient variant of SL1344 | (1) |
| *E. coli* DH5α | Cloning strain | NEB |
| *S.* Typhimurium SL1344 Δ*aroA* | *aroA* null deletion in SL1344 | This study |
| *S.* Typhimurium SL1344 Δ*ycaL* | *ycaL* null deletion in SL1344 | This study |
| **Plasmids** |  |  |
| pBAD18 | Arabinose inducible over expression vector | ATCC |
| pQE60*ndeI* | T5 IPTG inducible vector | (2) |
| pQE60-aroA_his | *aroA* cloned into pQE60nedI encoding a C-terminal poly histidine tag. | This study |
| pQE60-ycaL-his | *ycaL* cloned into pQE60nedI encoding a C-terminal poly histidine tag. | This study |
| pQE60-aroA_ycaL-his | *aroA-ycaL* cloned into pQE60nedI encoding a C-terminal poly histidine tag. | This study |
| pBAD18-aroA-his | His-tagged AroA subcloned into pBAD18 using the *EcoRI* and *HindIII* sites. | This study |
| pBAD18-ycaL-his | His-tagged ycaL subcloned into pBAD18 using the *SalI* and *HindIII* sites. | This study |
| pBAD18-aroA-ycaL_his | AroA and His-tagged YcaL subcloned into pBAD18 using the *EcoRI* and *HindIII* sites. | This study |
| pLUX | Reporter vector containing bacterial luminescence gene operon. | (3) |
| pLUX-p*nrdA* | *Salmonella* *nrdA* promoter cloned between the *XhoI* and *BamHI* sites. | This study |
| pLUX-p*nrdD* | *Salmonella* *nrdD* promoter cloned between the *XhoI* and *BamHI* sites. | This study |
| pLUX-p*nrdH* | *Salmonella* *nrdH* promoter cloned between the *XhoI* and *BamHI* sites. | This study |
| pLUX-p*gyrA* | *Salmonella* *gyrA* promoter cloned between the *XhoI* and *BamHI* sites. | This study |
| pKD4 | Template for amplification of the *aph* kanamycin resistance cassette. | (4) |
| pKD46 | Plasmid encoding λ-red recombinase, temperature sensitive. | (4) |
| pCP20 | Plasmid encoding FLP recombinase, temperature sensitive. | (74) |

**References**

1. Hoiseth SK, Stocker BA. 1981. Aromatic-dependent *Salmonella* typhimurium are non-virulent and effective as live vaccines. Nature 291:238-9.

2. Raghunathan D, Wells TJ, Morris FC, Shaw RK, Bobat S, Peters SE, Paterson GK, Jensen KT, Leyton DL, Blair JM, Browning DF, Pravin J, Flores-Langarica A, Hitchcock JR, Moraes CT, Piazza RM, Maskell DJ, Webber MA, May RC, MacLennan CA, Piddock LJ, Cunningham AF, Henderson IR. 2011. SadA, a trimeric autotransporter from *Salmonella enterica* serovar Typhimurium, can promote biofilm formation and provides limited protection against infection. Infect Immun 79:4342-52.

3. Burton NA, Johnson MD, Antczak P, Robinson A, Lund PA. 2010. Novel aspects of the acid response network of *E. coli* K-12 are revealed by a study of transcriptional dynamics. J Mol Biol 401:726-42.

4. Datsenko KA, Wanner BL. 2000. One-step inactivation of chromosomal genes in *Escherichia coli* K-12 using PCR products. Proc Natl Acad Sci U S A 97:6640-5.
