# Supplementary material for: Genome-wide fitness analysis of Salmonella enterica reveals aroA mutants are attenuated due to iron restriction in vitro
Source: mBio. 2024 Sep 17;15(10):e03319-23. doi: 10.1128/mbio.03319-23 (PMC11481492; doi:10.1128/mbio.03319-23)
Supplement: Table S6 — Primers used in this study. [file mbio.03319-23-s0007.docx]

**Table S6.** Primers used in this study

| aroA_Check_F | CACGGCCAGTCTGTGGGGTTTTTATTTC |
| --- | --- |
| aroA_internal_F | TACGATCCTGGACCCTAAATGTAC |
| ycaL_internal_R | GGCCCAGTACGCCTTCAATTTCATTATC |
| aroA_GA_F | CAGAATTCATTAAAGAGGAGAAATTACATATGGAATCCCTGACGTTACAAC |
| aroA_histagged_GA_R | GCTTAGTGATGGTGATGGTGATGGGCAGGCGTACTCATTCGC |
| ycaL_GA_F | CAGAATTCATTAAAGAGGAGAAATTACATATGAAAAATAAATCATTACTACTGGCGGTGGCG |
| ycaL_histagged_GA_R | GCTTAGTGATGGTGATGGTGATGCTTTTTATCTTCGGCAATTCTGTCGCG |
| pQE60_ndeI_GA_R | CATATGTAATTTCTCCTCTTTAATGAATTCTGTG |
| pQE60_ndeI_GA_F | CATCACCATCACCATCACTAAGC |
| PQE60_check_F | CACACAGAATTCATTAAAGAGG |
| pQE60_check_R | CATTACTGGATCTATCAACAG |
| pBAD_seq_F | GCTATGCCATAGCATTTTTATCC |
| pBAD_seq_R | CGTTCTGATTTAATCTGTATCAGG |
| pLUX_seq_F | CCGACGTCTAAGAAACCATTATTATC |
| pLUX_seq_R | CACTAAATCATCACTTTCGGGAAAG |
| nrdA_promoter_XhoI_F | TTAACTCGAGTTCGATGAAGAAATCAGCATTCG |
| nrdA_promoter_bamHI_R | AACGGGATCCGTGGTTTTCTACCTGTTTACCTG |
| nrdH_promoter_XhoI_F | TTAACTCGAGACCGTCTGGCGATCGGTATGCG |
| nrdH_promoter_bamHI_R | AACGGGATCCTAAAATGAGAAAACGGCGCG |
| nrdD_promoter_xhoI_F | TTAACTCGAGATCTTTTACAAACGCTACAGC |
| nrdD_promoter_bamHI_R | AACGGGATCCGATCACTCCATATTTGTAAGAACAG |
| gyrA_Promoter_xhoI_F | TTAACTCGAGCCGAGTAACTCGCAGCCAAC |
| gyrA_promoter_bamHI_R | AACGGGATCCCTAACCGCTATCCCTCTACTGTATCC |
| ycaL_dln_pKD4 | ATAATAGTTATCAACGGAAGATTAGACACATTGATTTATGTGTTTTTTTGAGCGATTGTGTAGGCTGGAG |
| ycaL_dln_pKD4 | AGAAACCAGCCCGAAAGATGACAATGATTAGCGGGCCGGCGGCCATGGTCCATATGAATATCCTCCTTAG |
| aroA_dln_F | CACGGCCAGTCTGTGGGGTTTTTATTTCTGTTTTTTGAGAGTTGAGTTTCATTGTGTAGGCTGGAGCTGC |
| aroA_dln_R | GACTCGGCGCGCCAGCCCGTCGACTGGCGCAACAGAAGACGGTCCATATGAATATCCTCCTTAGTTCCTAT |
| ycaL_check_F | CGATCCTGGACCCTAAATGTAC |
| ycaL_check_R | ACCGCTTGGGCCATCAA |
